# Microclimate refugia shape microclimatic niches and predict individual variability in post‐breeding migration in a partially migratory species

**DOI:** 10.1111/1365-2656.70147

**Published:** 2025-09-28

**Authors:** Rita F. Ramos, Karolina Zalewska, James J. Gilroy, João P. Silva, Aldina M. A. Franco

**Affiliations:** ^1^ School of Environmental Sciences University of East Anglia Norwich UK; ^2^ CIBIO/InBIO, Centro de Investigação em Biodiversidade e Recursos Genéticos, Laboratório Associado Universidade do Porto, Campus Agrário de Vairão Vairão Portugal; ^3^ CIBIO/InBIO, Centro de Investigação em Biodiversidade e Recursos Genéticos, Laboratório Associado Instituto Superior de Agronomia, Universidade de Lisboa Lisbon Portugal; ^4^ Departamento Biologia, Faculdade de Ciências Universidade do Porto Porto Portugal; ^5^ BIOPOLIS Program in Genomics, Biodiversity and Land Planning CIBIO, Campus de Vairão Vairão Portugal; ^6^ Estação Biológica de Mértola (EBM) Mértola Portugal

**Keywords:** microclimatic niche, movement strategy, niche tracking, partial migration, seasonal movements, seasonal niche

## Abstract

1. The characterization of species' environmental niches can help predict biodiversity responses to global environmental change and identify areas where environmental suitability declines as the conditions change. However, environmental niches, that is the full range of conditions a species experiences, are frequently described at coarse spatial and temporal scales, thus are unlikely to capture the across‐individual variability in exposure to microclimate conditions. Within species ranges and even within populations, individuals may vary in their ability to access microclimate refugia or may adopt different movement strategies to avoid exposure to unsuitable conditions. This individual variability currently remains unclear but could help us understand species' capacity to adjust to changes in climate.
2. We used an 11‐year satellite tracking dataset and high‐resolution remotely sensed habitat and climate information to investigate the microclimatic niche of a partially migratory grassland bird, the endangered little bustard (*Tetrax tetrax*) in the species' western stronghold populations in Southern Europe. Our study, including both breeding and post‐breeding seasons, aimed to determine whether the local conditions experienced by individuals during the breeding season can be used to predict individual movement strategies after breeding. Furthermore, we examined whether the distance travelled during post‐breeding dispersive migration influenced the level of dissimilarity between seasonal niches experienced by individuals.
3. The little bustard microclimatic niche was characterized along a gradient of temperature and microclimate refugia availability. Our results revealed that individuals occupying breeding areas with low microclimate refugia availability were more likely to move longer distances after breeding. Furthermore, long‐distance migratory individuals maintained similar microclimatic niches across seasons, whereas short‐distance migrants predominantly displayed a higher niche dissimilarity between seasons.
4. Temperature and microclimate refugia availability during the breeding season can help predict individual differences in migratory behaviour of little bustards and their niche dissimilarity across seasons.
5. Global warming and subsequent declines in microclimate refugia availability may force this species to move earlier and travel longer distances after breeding. This study provides information that can help design conservation strategies for little bustards and other endangered grassland bird species exposed to high temperatures.

## Graphical abstract

The niche is characterized along a gradient of temperature and microclimate refugia. Individuals in breeding areas with low microclimate refugia availability are more likely to cover larger distances to post‐breeding areas, increasing exposure to risks. Additionally, migratory individuals maintain consistent niches across seasons, while resident and short‐distance migrants switch niches.

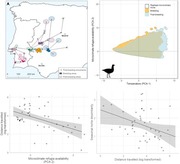

## Sumario

1. A caracterização dos nichos ambientais das espécies pode ajudar a prever as respostas da biodiversidade às alterações ambientais globais e a identificar áreas onde a adequação ambiental diminui à medida que as condições mudam. No entanto, os nichos ambientais, ou seja, todas as condições que afetam uma espécie, são frequentemente descritos em escalas espaciais e temporais grosseiras, pelo que é improvável que captem a variabilidade entre indivíduos na exposição às condições microclimáticas. Nas áreas de distribuição das espécies e mesmo dentro da mesma população, os indivíduos podem variar na sua capacidade de aceder a refúgios microclimáticos ou podem adotar diferentes estratégias de movimento para evitar a exposição a condições inadequadas. Esta variabilidade individual é ainda pouco estudada, mas pode ajudar‐nos a compreender a capacidade das espécies se adaptarem às alterações climáticas.
2. Utilizámos um conjunto de dados de seguimento por satélite de 11 anos e informações de alta resolução sobre o habitat e o clima obtidas através de sensoriamento remoto para investigar o nicho microclimático de uma ave estepária parcialmente migratória e em perigo de extinção, o sisão (*Tetrax tetrax*), nas populações ocidentais da espécie, no sul da Europa. O nosso estudo, que incluiu as épocas de reprodução e pós‐reprodução, teve como objetivo determinar se as condições locais a que os indivíduos estão expostos durante a época de reprodução podem ser utilizadas para prever as estratégias de movimento individuais após a reprodução. Examinámos ainda se a distância percorrida durante a migração de pós‐reprodução influencia o nível de dissimilaridade entre os nichos sazonais de diferentes indivíduos.
3. O nicho microclimático do sisão foi caracterizado ao longo de um gradiente de temperatura e disponibilidade de refúgio microclimático. Os nossos resultados revelaram que os indivíduos que ocupavam áreas de reprodução com baixa disponibilidade de refúgios microclimáticos eram mais propensos a percorrer distâncias maiores após a reprodução. Além disso, indivíduos que migravam longas distâncias mantiveram nichos microclimáticos semelhantes ao longo das duas épocas, enquanto os migradores de curta distância apresentaram predominantemente uma maior dissimilaridade de nichos entre épocas.
4. A temperatura e a disponibilidade de refúgios microclimáticos durante a época de reprodução podem ajudar a prever diferenças individuais no comportamento migratório dos sisões e a sua dissimilaridade de nichos entre as épocas.
5. O aquecimento global e o consequente declínio na disponibilidade de refúgios microclimáticos podem forçar esta espécie a deslocar‐se mais cedo e a percorrer distâncias mais longas após a reprodução. Este estudo fornece informações que podem ajudar a desenhar estratégias de conservação para sisões e outras espécies estepárias ameaçadas de extinção que estão expostas a altas temperaturas.

## 1 INTRODUCTION

A species' niche can have multiple interconnected dimensions, including environmental, ecological and biogeographic factors (Soberón, 2007). The combination of all niche dimensions limits species distributions to a fraction of the fundamental niche—the realized niche (Hutchinson, 1978). The extent of any given niche dimension may vary between individuals and across populations due to individual variability in physiological, biological and behavioural traits (Soberón, 2007). Some individuals may be niche specialists, having a narrow niche relative to their population or species, while others may be generalists with relatively broad niches (Bolnick et al., 2003). The occurrence of both specialist and generalist individuals within populations is a source of species diversity (Araújo et al., 2011; Carlson et al., 2021) that may allow species to better adjust to environmental changes (Bolnick et al., 2007). Understanding the underlying factors affecting individual variability and its demographic consequences can aid in the design of targeted conservation measures aiming to help species adapt to the current fast rate of environmental change.

Species and individual niches may also vary throughout the annual cycle induced by seasonal differences in environmental conditions (Cohen & Jetz, 2023; Winger et al., 2018), especially at higher latitudes. Through migration, individuals can either maintain their environmental niche throughout the year by moving between areas with similar environmental characteristics, a phenomenon referred to as ‘niche tracking’ (Gómez et al., 2016), or undergo a complete niche change, known as ‘niche switching’ (Ponti et al., 2020). These seasonal niches play a crucial role in individual fitness and survival, with the breeding season often being the most critical period (Harrison et al., 2011) when individuals may require access to specific habitats, food resources or social conditions (Ponti et al., 2020). In the post‐breeding period, individuals can be more mobile, utilizing different combinations of environmental conditions, and therefore have broader niches (Suárez‐Seoane et al., 2008). The movement strategies that enable niche tracking or switching can also vary between individuals within populations and species (Fandos et al., 2020; Illán et al., 2022).

Partial migration, where some individuals within a population migrate, while others remain resident at their breeding sites throughout the year, is more common than previously thought (Buchan et al., 2020; Chapman et al., 2011; Newton, 2010; Reid et al., 2018). For the two strategies (residency and migration) to be maintained within a population, both need to yield similar fitness or relative benefits (Buchan et al., 2020; Lundberg, 1988). Migration may have a higher cost than residency, as migratory individuals, especially those performing long‐distance movements, face high energetic costs and an increased probability of encountering threats both during migration and at post‐breeding sites (Alerstam et al., 2012; Buchan et al., 2023; Wikelski et al., 2003). However, moving also allows individuals to access new resources and maintain their niche, while residents and short‐distance migrants may be exposed to seasonal changes and unsuitable conditions at their year‐round sites, and consequently occupy different niches across their annual cycle (Alerstam et al., 2012). The mechanisms and drivers underlying between‐individual variability in movement behaviour and niche tracking strategies are still poorly understood (but see Illán et al., 2022) or have mostly been examined at coarse scales (Fandos et al., 2020; Zurell et al., 2018) but have a wide interest when planning conservation measures.

The emergence of microclimate and high‐resolution environmental data, including information at the scale at which individuals experience their environments (Carlson et al., 2021; Suggitt et al., 2018), is opening new research opportunities and increasingly being used to understand responses to environmental change (Maclean & Early, 2023; Massimino et al., 2020; Potter et al., 2013) and variability in individual behaviour (Ramos et al., 2023b). This has been facilitated by recent advances in animal tracking technologies (e.g. Nathan et al., 2022), increased availability of remotely sensed habitat information (Pettorelli et al., 2005; Valerio et al., 2020) and lower costs of cloud computing, which were major limitations just a few years ago (Schulte to Buhne & Pettorelli, 2017).

In this study, we analyse movement data of 46 little bustards (*Tetrax tetrax*), satellite tracked over 11 years, in five Southern European breeding populations and characterize individual and population realized microclimatic niches, characterized using the microclimatic temperature and refugia conditions that are utilized across the breeding and post‐breeding seasons. We determine the micro‐scale environmental factors (e.g. temperature, microclimate refugia and food availability) that may influence individual migratory distances and subsequent seasonal niche dissimilarities of this partially migratory species. We hypothesize that (1) increased access to microclimate refugia and food availability within breeding areas reduces post‐breeding migratory distances, since access to temperature refugia sites and foraging resources is expected to be available locally; (2) individuals exposed to higher temperatures during the breeding season will undergo longer post‐breeding dispersive migrations as physiological limits may be reached at the breeding sites, forcing individuals to move; and (3) individuals moving relatively shorter distances will have more dissimilar breeding and post‐breeding niches than those dispersing farther from the breeding areas. This is expected because individuals moving longer distances may be able to find optimal microclimate and foraging conditions (similar to the breeding areas), while those moving shorter distances may be using local refugia in suboptimal sites but do not experience the cost of moving to new areas.

## 2 MATERIALS AND METHODS

### 2.1 Study site and target species

The Iberian Peninsula is simultaneously a global biodiversity hotspot and one of the world's most vulnerable regions to climate change (Pörtner et al., 2022). The region is expected to suffer from extensive warming and increasing drought frequency in the near future (Jones et al., 2020), which is expected to cause habitat changes, species range contractions or even (local) extinctions (Pörtner et al., 2022). Conditions are particularly rough in flat and open areas with low vegetation cover, such as semi‐natural grasslands, where species are exposed to high temperatures throughout most of the year. Within the Iberian Peninsula, semi‐natural grasslands are one of the most climate change‐sensitive habitats, due to their ecological characteristics and dependency on human management through agricultural activities and livestock grazing (Emanuelsson, 2008).

Semi‐natural grasslands in Iberia are crucial for grassland birds, including several endangered species that use this habitat mainly during the breeding period. Among those species is the little bustard, *Tetrax tetrax* (Linnaeus, 1758), which is a medium‐sized grassland specialist bird classified as ‘Near Threatened’ (BirdLife International and Handbook of the Birds of the World, 2021). Recent studies indicate a severe decline in little bustard breeding numbers in the Iberian Peninsula (Morales & Bretagnolle, 2022; Silva et al., 2023), which used to be a stronghold of this species' western distribution (García de La Morena et al., 2018; Silva et al., 2018). The main Iberian breeding populations are predominantly concentrated in the Extremadura, Castilla La Mancha (Spain), and Alentejo (Portugal) regions (Equipa Atlas, 2008; García de La Morena et al., 2018), all of which are affected by elevated temperatures and are vulnerable to climate change (Pörtner et al., 2022; Ramos et al., 2023a).

Little bustards are a partially migratory species (García de La Morena et al., 2015), where many individuals move to northern or coastal post‐breeding areas, where temperatures are milder and food availability is higher (García de La Morena et al., 2015; Silva et al., 2007). These post‐breeding movements occur between June and August and are mostly triggered by food shortages, increased levels of exposure to high temperatures and a lack of microclimate refugia in the breeding areas (Ramos et al., 2023b). This species is known to decrease its activity patterns at temperatures above 25°C (Silva et al., 2015) and makes use of microclimate refugia sites during the warmer parts of the year (Ramos et al., 2023a).

### 2.2 Tracking and location data

Between 2009 and 2019, 46 male little bustards were captured and tagged in five distinct breeding areas across the southwest Iberian Peninsula, in Alentejo (Portugal) and Extremadura (Spain), during the breeding season (April and May). Breeding males were attracted by a stuffed female acting as a decoy and trapped with snares (Ponjoan et al., 2010; Ramos et al., 2023a). GPS tracking devices varying between 2% and 4% ($\bar{x}$ = 3.2%) of the birds' mass (Kenward, 2000) were deployed using a thoracic harness made of Teflon Ribbon with a weak link to avoid lifelong deployment. Two types of solar GPS devices were used. Platform Transmitter Terminal (Solar Argos/GPS 30g PTT—Microwave Telemetry) devices were deployed on 19 birds between 2009 and 2011, and Global System for Mobile Communications (GSM) devices (Flyway 38g—Movetech Telemetry) were deployed on 28 birds between 2014 and 2019. Transmitters were programmed to record a GPS position every 2 h (PTT) or 10–30 min (GSM).

For this work, no ethical approval was required. Bird trapping and the deployment of GPS devices were approved by the Instituto da Conservação da Natureza e das Florestas (Portuguese Government agency responsible for Wildlife and Forests Management and Conservation) through licences to João Paulo Silva (ICNF/CAPT/2014, ICNF/CAPT/2015) and Consejería de Medio Ambiente y Rural, Políticas Agrarias y Territorio of the Junta de Extremadura (Spanish Ministry of Environment and Rural, Agrarian Policies and Territory of the Extremadura region) through the licence to José Mª Abad‐Gómez.

We filtered the GPS locations dataset to only include locations on the ground with null velocity. Moreover, we included only daylight locations collected between 6 AM and 5 PM (breeding) and 7 AM and 8 PM (post‐breeding) to avoid capturing nocturnal roost sites. Only individuals captured before 1st of May and with at least 7 days of data prior to departure from the breeding areas were included in the analysis. This threshold was defined to reduce the influence of the tagging date, which can vary greatly between individuals, as well as to avoid characterizing the breeding season based on a relatively low sample size from individuals captured later in the season. As a result, for consistency, we defined the breeding season as starting from 1st of May for all individuals. The end of breeding and the start of the post‐breeding season was the date when each tracked little bustard left the breeding area and moved to a non‐breeding area for a minimum period of 30 days. Birds that used the breeding area during the whole year were considered residents (Jiguet & Bretagnolle, 2001), and the post‐breeding season was defined as their period between 15th of July and 15th of September. This represents the hottest period of the year in the Iberian Peninsula, when little bustards are exposed to extreme temperatures, as well as a period of food shortage (Ramos et al., 2023a; Silva et al., 2007).

### 2.3 Environmental data

We obtained micro‐scale environmental data for all little bustard GPS locations as well as within a 500 m buffer around each GPS location. Hourly temperatures were extracted at a 30 × 30 m resolution at 20 cm above‐ground (Ramos et al., 2023a) using the *microclima* (Maclean et al., 2019) and *NicheMapR* (Kearney & Porter, 2017) packages in R version 4.1.0 software (R Core Team, 2016). We used the fully automated microclimate model, which generated temperatures at the 30‐m scale while accounting for terrain characteristics, habitat type and vegetation information (such as canopy and shading effects). Within the buffer around each GPS location, we also calculated the minimum, maximum, mean, median and standard deviation of the hourly temperature.

Microclimate refugia are small patches within the broader landscape with features that may provide shelter from unfavourable, particularly high temperatures (Rull, 2009). For the studied species, microclimate refugia are characterized by small patches of non‐herbaceous vegetation, usually trees and shrubs, in an herbaceous matrix, which creates a more heterogeneous thermal landscape (Ramos et al., 2023a).

Previous studies have highlighted the importance of the availability of such sites for the little bustard (Ramos et al., 2023a, 2023b). Hence, we calculated two metrics—refugia availability and refugia use—and included them in subsequent analyses. Refugia use was calculated as the difference between the estimated temperature at the GPS location and the median temperature of the 500 m buffer area, while refugia availability was defined by the difference between the minimum and the median temperature within the buffer (Ramos et al., 2023a):

$$\begin{array}{cc} \text{REFUGIA}\;\mathrm{USE} & =\text{Temperature}\;\mathrm{GPS}\;\text{Location}\\ & -\text{Buffer Median Temperature} \end{array}$$

$$\begin{array}{cc} \text{REFUGIA AVAILABILITY} & =\text{Buffer Minimum Temperature}\\ & -\text{Buffer Median Temperature} \end{array}$$

Greater negative values of both variables indicate more microclimate refugia use and availability (Ramos et al., 2023a), since it translates to point temperatures cooler than the median of the buffer.

We also extracted the normalized difference vegetation index (NDVI) values for all little bustard GPS locations. Satellite‐derived NDVI is a measure of vegetation greenness and biomass, which is widely used to examine patterns of vegetation productivity and biodiversity distribution at large spatial and temporal scales (Pettorelli et al., 2005). As green plants are an important component of the little bustard's diet, especially during the breeding season, NDVI has been broadly used as a predictor of food availability (Pettorelli et al., 2005). Moreover, precipitation acts as a stimulus for vegetation growth, and therefore is highly correlated with NDVI (Li et al., 2019; Schultz & Halpert, 1993). Thus, NDVI is both a good indicator of precipitation patterns in the previous months (Pettorelli et al., 2005) and a proxy for food availability, which is a key determinant of little bustard movements and space use (Ramos et al., 2023b). NDVI was extracted from MODIS satellite imagery at a 250 m resolution and 8‐day interval, using Google Earth Engine (Didan, 2015; Gorelick et al., 2017). We evaluated all images retrieved to ensure that their quality was sufficient for use in the study (Didan, 2015). We calculated the average NDVI of the last 8 days before an individual departed its breeding area (or the end of the individual's breeding season). This was done to account for the immediate conditions an individual may be experiencing before leaving the breeding area.

### 2.4 Micro‐climate and the realized microclimatic niche

The little bustard's realized microclimatic niche was characterized using the principal component analysis (PCA‐occ) approach outlined by Broennimann et al. (2012). This method transforms the correlated environmental variables into independent principal components, generating a multidimensional shape representing the microclimatic niche. The number of dimensions considered was determined by the number of principal components cumulatively explaining over 70% of the variance within the initial dataset. The PCA‐occ was calculated using the *ade4* package (Dray & Dufour, 2007) and the *ecospat* (Broennimann et al., 2014) R packages, and using the temperature‐ and refugia‐related variables outlined above for all little bustard's GPS location data included in this study (Table 1).

**TABLE 1:** Description and spatial resolution of the micro‐scale temperature and microclimate refugia variables included in the principal component analysis (PCA‐occ), used to characterize the little bustard's realized microclimatic niche.

Variable	Description
Point temp.	Temperature at the GPS location (°C), measured at 30 m scale and 20 cm above‐ground
Mean temp.	Mean temperature within the 500 m buffer around the GPS location (°C), measured at a 30‐m scale
Median temp.	Median temperature within the 500 m buffer around the GPS location (°C), measured at a 30‐m scale
Max. temp.	Maximum temperature within the 500 m buffer around the GPS location (°C), measured at a 30‐m scale
Min. temp.	Minimum temperature within the 500 m buffer around the GPS location (°C), measured at a 30‐m scale
Temp. Std	Temperature standard deviation within the 500 m buffer around the GPS location (°C), measured at a 30‐m scale
(Microclimate) refugia use	Difference between temperature at GPS location and median temperature of the 500 m buffer
(Microclimate) refugia availability	Difference between the minimum temperature of the 500 m buffer and the median temperature of the 500 m buffer, for the GPS location

The PCA scores corresponding to observations belonging to each individual, population and season, both across the entire study period and for each year, were extracted. The microclimatic space (hereafter designated the realized microclimatic niche or microclimatic niche) utilized by little bustards in different parts of the range and annual cycle was determined by estimating the 99.5% bivariate normal kernel (to eliminate outliers) of the corresponding PCA scores on the retained first two principal components, using the package *adehabitatHR* (Calenge, 2006). This produces a two‐dimensional representation of the microclimatic niche. We then overlapped each population's breeding and post‐breeding niches with the total species' breeding and post‐breeding niche, respectively. We also calculated the percentage of overlap of the seasonal niches across all populations, as well as between the seasonal niches and the overall little bustard niche. At the individual level, the overlaps were carried out separately for each population and each year that a given individual was tracked for, that is the seasonal niche of any individual in any year was overlapped with the niche of the same year and season of the population that individual belonged to. Finally, we determined, for each individual, the percentage of the total seasonal microclimatic niche occupied.

### 2.5 Statistical analysis

Adult little bustards tend to have a strong site fidelity in both breeding and post‐breeding seasons (Alonso et al., 2020; García de La Morena et al., 2015), which can be derived from a learning process while they are immature birds, as documented in other bustard species (Burnside et al., 2017; Newton, 2010). Moreover, previous studies showed that the distance travelled during the post‐breeding movements tends to be relatively consistent between years for the same individual (Ramos et al., 2023b). Since little bustard age is difficult to estimate, for birds with multiple years of data, only data from the last, most recent year of tracking were used in the analyses below. By including only the most recent year of data for each individual, we maximize the chance of capturing the consistent behaviour that characterizes adult individuals.

We obtained the centroids of each individual's daily utilization distribution (calculated as the 90% bivariate normal kernel of the individual's relocations). For the breeding season, we calculated the mean centroid across each individual's daily utilization distributions of that season. The distance travelled by the individual to the post‐breeding areas was calculated as the Euclidean distance between the individual's mean breeding centroid and the centroids of each daily post‐breeding utilization distribution (for more details, see Ramos et al., 2023b). The mean centroid for the breeding distribution was used because, within a season, individuals consistently use the same areas; however, during the post‐breeding season some individuals may move and use more than one area, and therefore, the movement distance was calculated for each daily utilization distribution during the post‐breeding. The distances calculated were then summed to obtain the total distance travelled by each individual.

We obtained the centroids of the individual breeding and post‐breeding microclimatic niches and calculated the Euclidean distance between them for each individual. This distance represents the dissimilarity between individual seasonal niches, where higher values mean more dissimilar niches.

We fitted two linear mixed‐effects models using the *lme4* package (Bates et al., 2014). The first model analysed the influence of the individual breeding niche on the dispersive migration distance travelled, by using the coordinates of the centroids of each individual's breeding microclimatic niche (scaled) and the average NDVI of the last 8 days before departure from the breeding area (or last 8 days of the breeding season), to explain the distance travelled (natural logarithm‐transformed). The second model examined the effect of the individual used niche as well as the distance travelled on the dissimilarity between seasonal niches. To analyse this, we used the dissimilarity between each individual's breeding and post‐breeding niche (calculated as the distance between niche centroids, see above) as a response variable, and included the percentage of overlap between the individual's breeding niche and the breeding niche of its breeding population in the same year, the scaled *x* (temperature) and *y* (microclimate refugia availability) coordinates (in PC units) of the centroids of each individual's breeding microclimatic niche, as well as the distance travelled by that individual during its dispersive migration (natural logarithm‐transformed). In both models, we included the breeding population as an independent random intercept effect.

Some populations had a relatively small number of tracked individuals, and this low sample size could potentially bias our results. To test whether a lower sample size is sufficient to produce an adequate representation of the population niche, we randomly selected five individuals from populations with a larger number of tracked individuals (Merida, Vila Fernando and Castro Verde) and repeated the seasonal niche overlap analysis only using data for the selected five individuals. We repeated this process for each possible combination of five individuals within each population (up to 10,000 combinations; see Supporting Information S3 for details). We then compared the population seasonal niches produced using all data with those obtained using data from only five individuals and found that the extent of seasonal niche overlap obtained for populations consisting of only five individuals matches the seasonal niches produced with all available data. This suggests that five individuals may be a representative sample of the populations included in this study.

## 3 RESULTS

**FIGURE 1:**
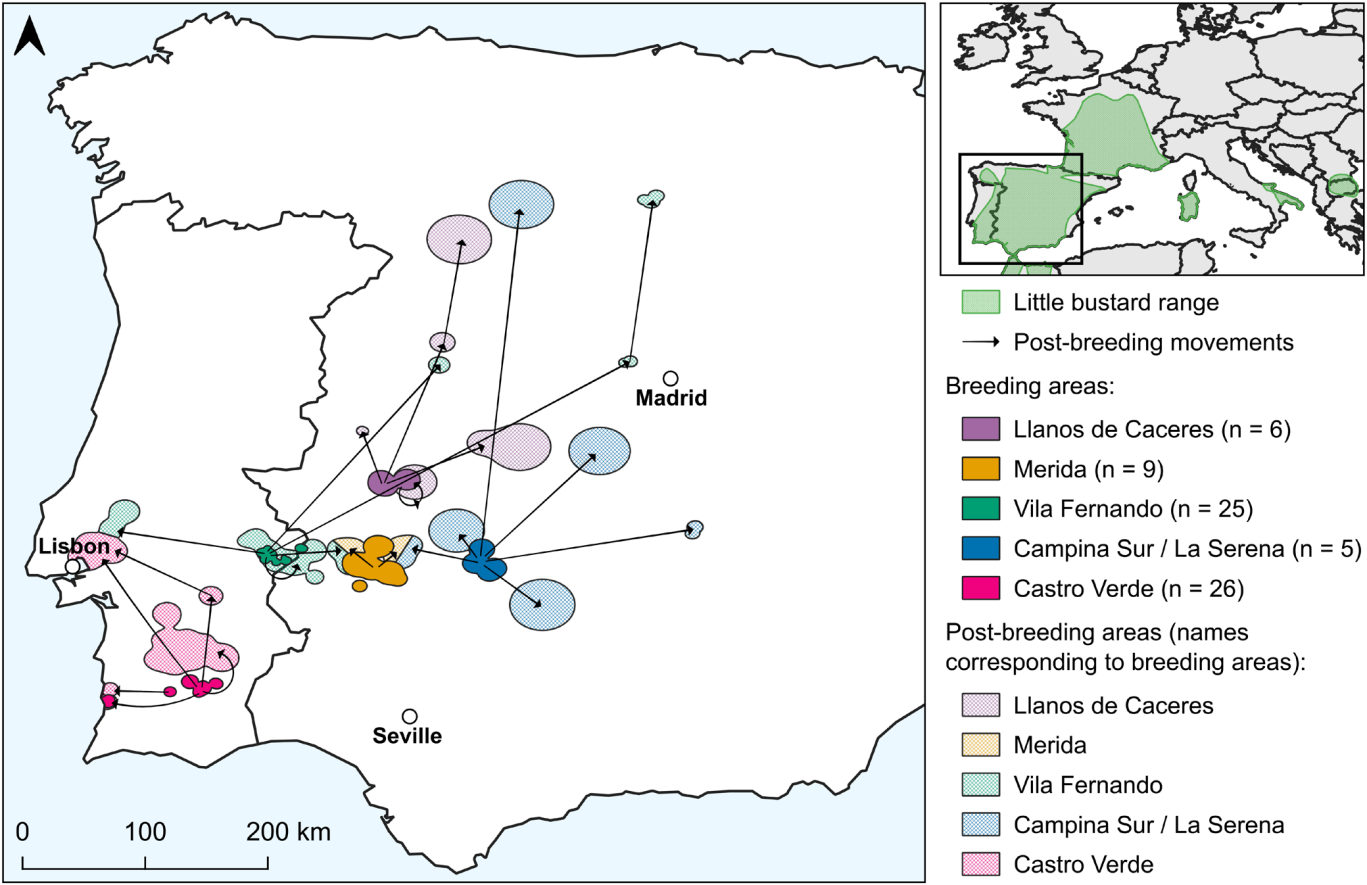
Migratory movements of 46 male little bustards from breeding (coloured) to post‐breeding (hollow) areas in the Iberian Peninsula obtained from GPS tracking data. Each colour represents a breeding population, and the number of individual/years tracked within each population is shown in parentheses (total of 71 individual/years). The arrows show movements to post‐breeding areas. The little bustard's range within Europe (BirdLife International and Handbook of the Birds of the World, 2021) is shown in green in the inset map.

During the 11‐year study, we captured and deployed GPS tracking devices on 46 male little bustards in five different populations. We collected more than 30,000 GPS locations in both breeding and post‐breeding seasons, which we used to build the high‐spatial resolution realized microclimatic niche. The breeding areas used by the little bustards varied between 36,123 ha in Vila Fernando and 162,547 ha in Merida (Figure 1, Table 2). The distance travelled to the post‐breeding sites varied greatly between individuals, with movements ranging from 1 to 421 km (median = 36 km), and with some birds using more than one post‐breeding area (Figure 1).

**TABLE 2:** General spatial (areas used, total number of individuals and GPS locations) and realized microclimatic niche (overlap of breeding and post‐breeding season microclimatic niches) information for each breeding population.

Capture site	Breeding used area (ha)	Num. ind.^a^	Num. GPS locations^a^	Breeding season overlap^b^	Post‐breeding season overlap^b^
Llanos de Caceres	77,846	6	1728	35.83	35.13
Merida	162,547	9	7197	98.15	97.14
Vila Fernando	36,123	25	9931	96.49	94.27
Campina Sur/La Serena	91,923	5	3170	47.00	37.22
Castro Verde	57,996	26	8296	44.52	78.30

^a^ Number of individual/years in relation to the overall breeding season microclimatic niche.

^b^ Percentage of overlap with the post‐breeding season's overall microclimatic niche.

### 3.1 Realized microclimatic niche

**FIGURE 2:**
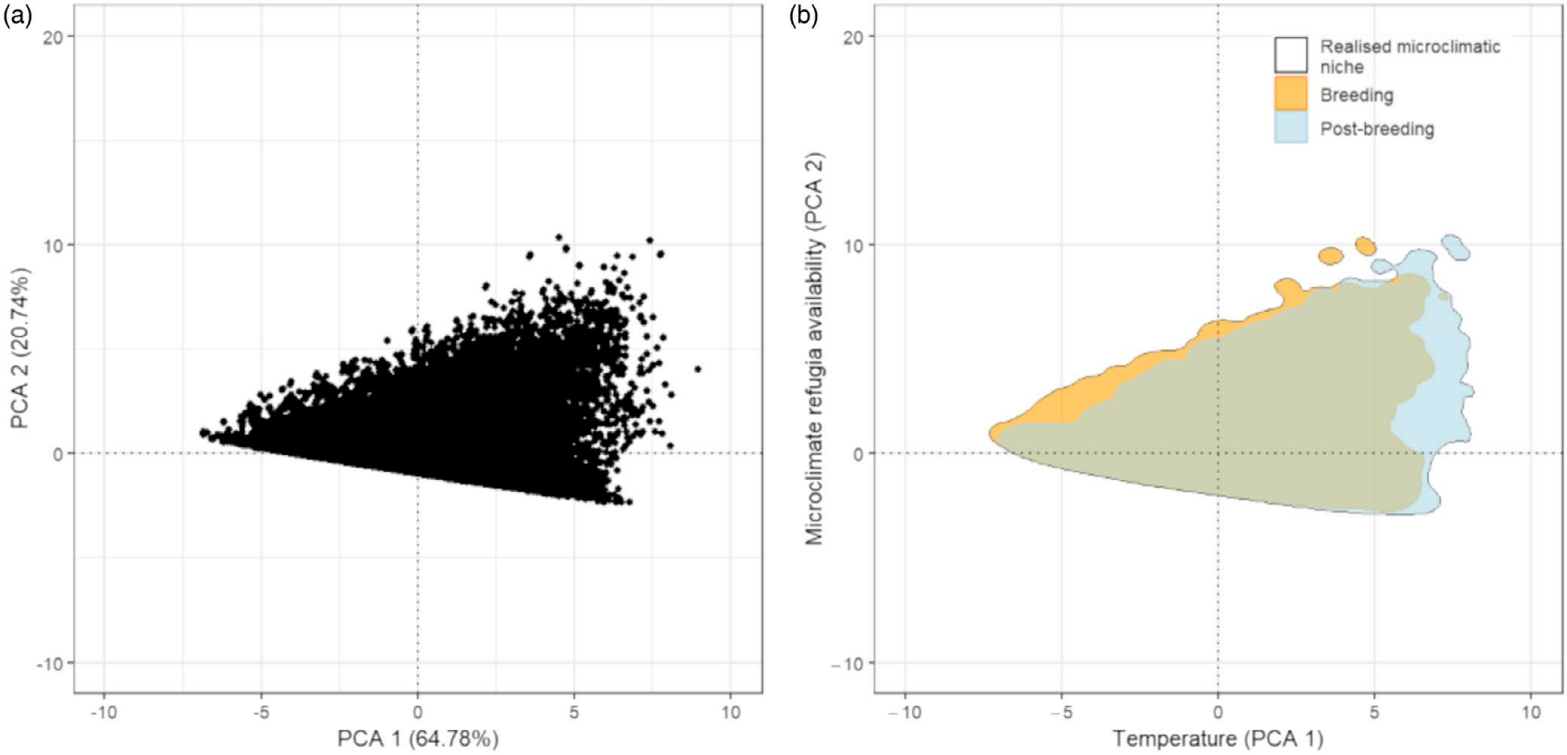
(a) Principal component scores representing the little bustard‐realized microclimatic niche determined based on GPS locations obtained in the Iberian Peninsula. (b) Realized microclimatic niche during the breeding (orange) and post‐breeding (blue) seasons.

The little bustard realized microclimatic niche was characterized by the first two PC axes, capturing 85.53% of the variation in the data (64.78% by the first and 20.74% by the second component, Figure 2a). The first axis was strongly and positively associated with temperature (point temperature, mean temperature, median temperature, minimum temperature and maximum temperature). The second principal component had a strong positive association with increasing refugia availability and temperature standard deviation (Supporting Information S1).

The breeding season niche represented 85.72% of the total realized microclimatic niche, while post‐breeding represented 91.00% (Figure 2b). The post‐breeding niche overlapped in 89.55% with the breeding niche (Figure 2b). Overall, the post‐breeding niches were characterized by higher temperatures with more microclimate refugia availability compared to the breeding niches.

Out of the five breeding populations tracked in this study, three had a breeding niche smaller than 50% of the overall used niche (Campina Sur/La Serena, Castro Verde and Llanos de Caceres) (Table 2, Supporting Information S2). The two other breeding populations (Merida and Vila Fernando) had a wide breeding niche, representing more than 95% of the season's used niche (Table 2, Figure 2).

**FIGURE 3:**
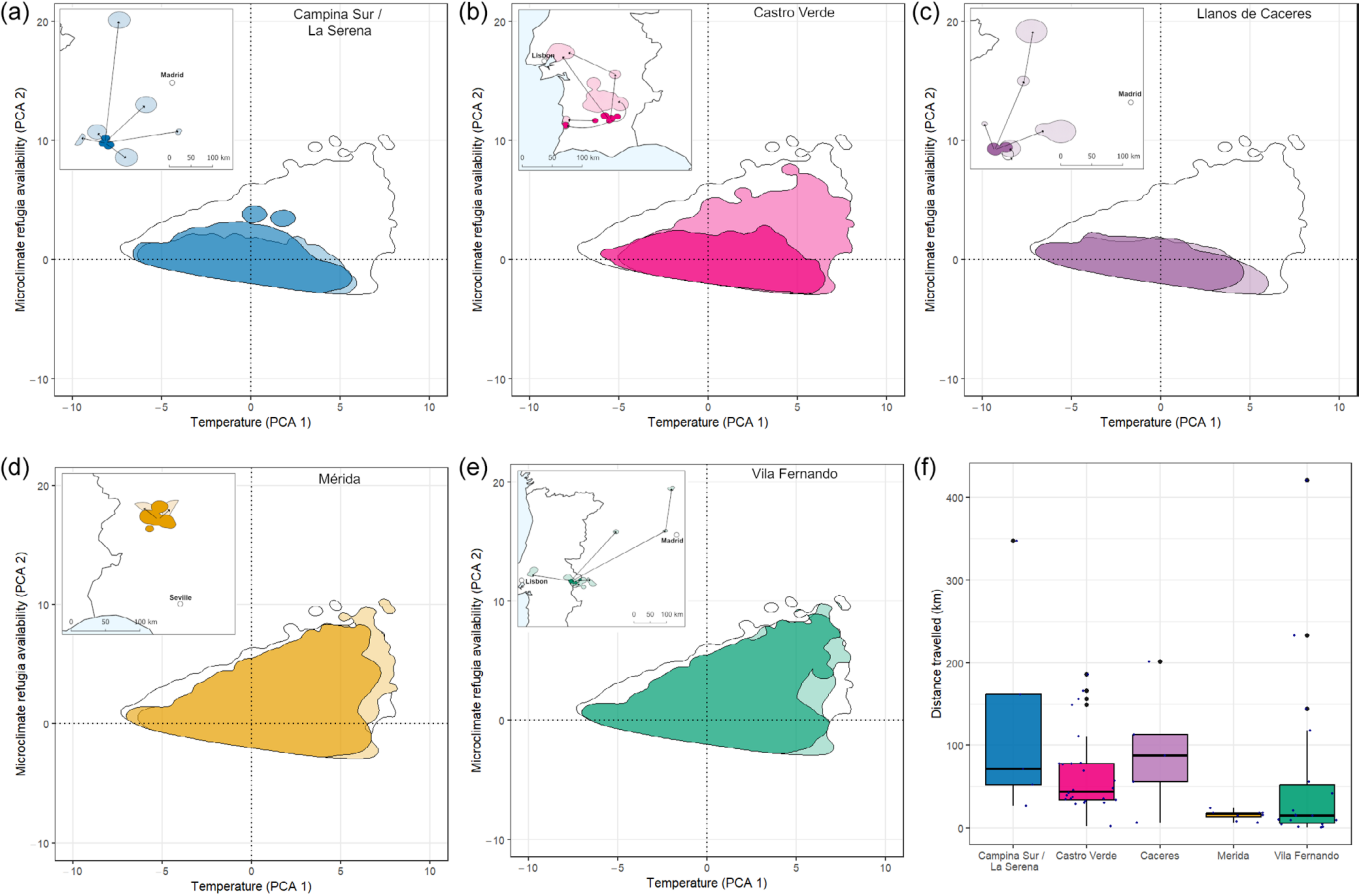
Breeding (full colour) and post‐breeding (hollow) microclimatic niche used by each breeding population with information of the post‐breeding locations and movements (inset map) (a–e), and the distances travelled (f). From top left to bottom right: Campina Sur/La Serena (a), Castro Verde (b), Llanos de Caceres (c), Merida (d) and Vila Fernando (e). Black outline shows the realized microclimatic niche across all populations and seasons. See Supporting Information S4 for details on each individual and population.

For most breeding populations, the microclimatic niche remained similar in both seasons, despite the post‐breeding movements performed by the individuals (Figure 3). The majority of populations maintained a similar percentage of niche occupied across seasons, except Castro Verde, for which the seasonal microclimatic niche increased from 44.52% of the overall species niche in the breeding season to 78.30% in the post‐breeding season (Table 2, Supporting Information S2 and S4), with the expansion occurring towards areas with more microclimate refugia availability (Figure 3b).

### 3.2 Distance travelled and niche dissimilarity in relation to the breeding microclimatic niche characteristics

**FIGURE 4:**
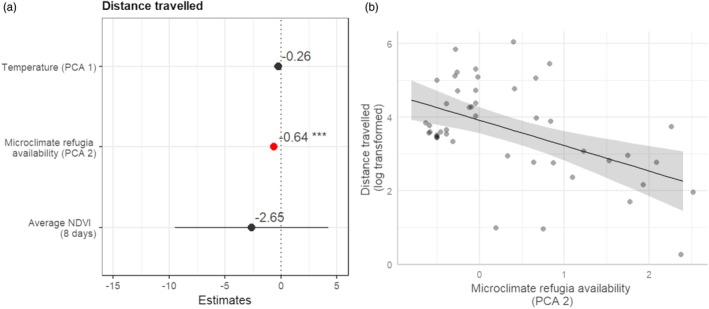
(a) Coefficient estimates, from linear mixed‐effects models, explaining the individual distance travelled using microclimatic niche dimensions (temperature (PCA1) and microclimate refugia availability (PCA2)) and NDVI (*R* ^2^ presented in Figure S5.1). Covariate significance is shown: ***p < 0.01; * 0.01 < *p* < 0.05; none/otherwise, *p* > 0.05; and (b) the relationship between distance travelled and microclimate refugia availability (PCA2).

Distance travelled was negatively associated with microclimate refugia availability (the PCA2; estimate = −0.638, SE = 0.168, *p* < 0.001), while there was no significant association with temperature (PCA1; estimate = −0.261, SE = 0.182, *p* = 0.158) or NDVI (estimate = −2.648, SE = 3.395, *p* = 0.440; Figure 4).

**FIGURE 5:**
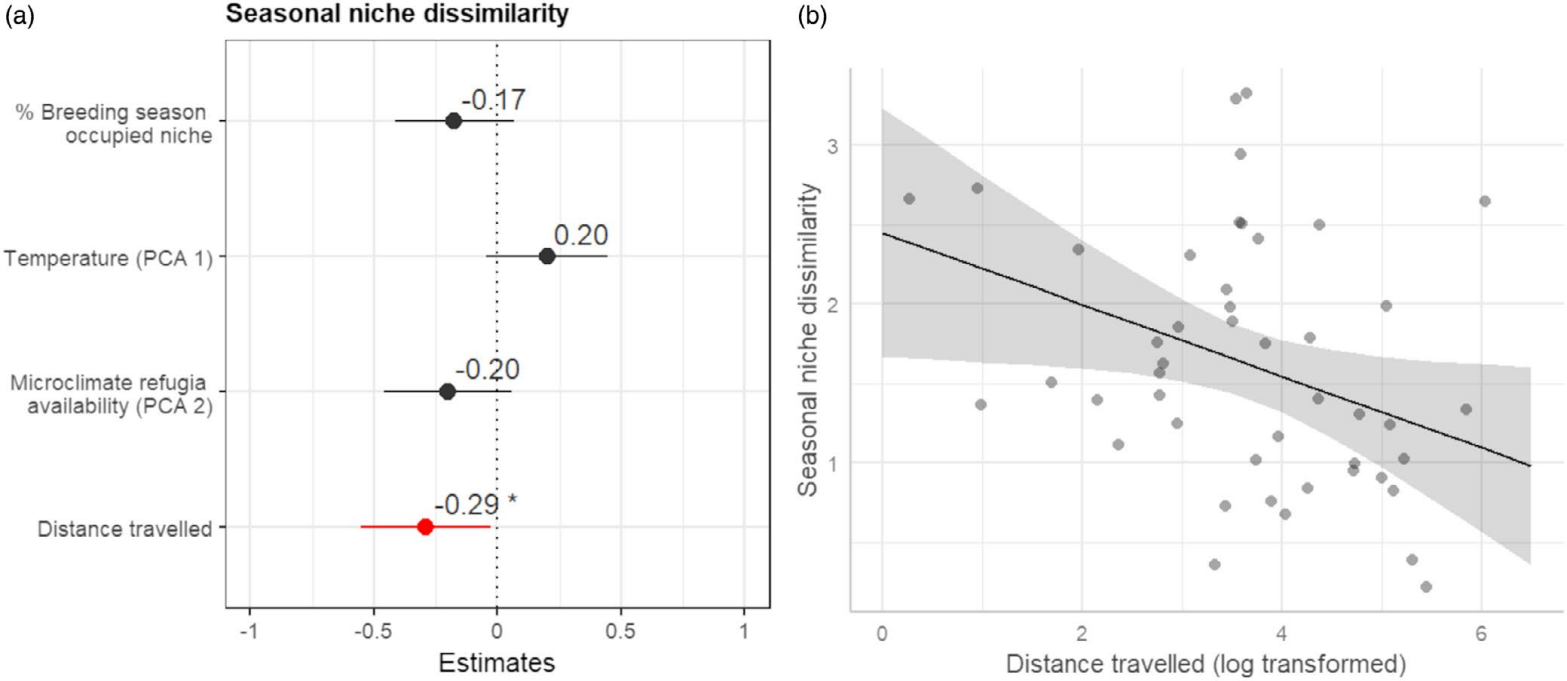
(a) Coefficient estimates from linear mixed‐effects models for the effect of niche characteristics and migratory distance travelled on niche dissimilarity between seasons (*R* ^2^ presented in Figure S5.2). Covariate significance is shown: ***p < 0.01; *0.01 < *p* < 0.05; none/otherwise, *p* > 0.05. (b) Dissimilarity between the seasonal niches in relation to the distance travelled in the post‐breeding dispersive migration.

Seasonal niches were more dissimilar as the distance travelled in the post‐breeding migration decreased (estimate = −0.289, SE = 0.130, *p* = 0.032), while there was no significant association with any microclimatic niche axis (PCA1 estimate = 0.200, SE = 0.122, *p* = 0.108; PCA2 estimate = −0.201, SE = 0.127, *p* = 0.122) or the individual's breeding microclimatic niche (estimate = −0.175, SE = 0.119, *p* = 0.149; Figure 5).

## 4 DISCUSSION

Our study, utilizing a robust dataset of 11 years of tracking data with more than 30,000 GPS locations, primarily focused on understanding the realized microclimatic niche of an endangered grassland bird specialist. Our study characterized the little bustard's microclimatic niche within the southern part of the Iberian Peninsula across gradients of temperature and microclimate refugia availability. While there was a significant overlap of the seasonal niches at the species level, some populations were restricted to smaller sections of the species' niche in the southern Iberian Peninsula. At the individual level, we observed a negative relationship between distance travelled and microclimate refugia availability during the breeding season, whereby individuals with less refugia available within their breeding sites tended to move longer distances. Additionally, distance travelled was also negatively related to niche dissimilarity between seasons, as birds that performed longer movements maintained a similar realized microclimatic niche across seasons.

Not surprisingly, temperature (thermal component of the niche) explained more than half of the variation within the data, representing the first axis of the niche. This finding is consistent with previous studies that have identified temperature as a critical factor in determining the macro‐scale environmental niches of species, including that of the little bustard (Delgado et al., 2011; Fandos & Tellería, 2020; Ponti et al., 2020). Temperature is a well‐known driver and limiting factor at both species and individual levels, frequently shown to have direct effects on individuals through thermal stress, as well as affecting them indirectly, by reducing food availability (Breed et al., 2013; Chen et al., 2011; Hao et al., 2012; Rastogi, 2007). Furthermore, extreme temperatures have also been linked to changes in individual breeding and migratory behaviour (Tomotani et al., 2018).

Microclimate refugia availability was the main variable defining the second dimension (component) of the microclimatic niche. Although little bustards only occasionally use microclimate refugia (Ramos et al., 2023a), these areas may become crucial during the post‐breeding season when temperatures increase and food becomes scarce (Silva et al., 2007). Areas of microclimate refugia availability can provide necessary shelter from high temperatures for both little bustards, as well as for arthropods, which are an important food source for this species, especially in drier habitats (González del Portillo et al., 2021; Jiguet, 2002; Suggitt et al., 2018). Given that little bustards show reduced activity levels when temperatures are above 25°C and can experience temperatures of up to 40°C during the post‐breeding season in the Iberian Peninsula (Ramos et al., 2023a; Silva et al., 2015), it is unsurprising that microclimate refugia availability may be an important component of the little bustard's realized microclimatic niche. Areas with microclimate refugia availability are usually characterized by small patches of shrubs and trees in a generally herbaceous matrix (Ramos et al., 2023a), creating a heterogeneous thermal landscape.

The population microclimatic niches varied mainly across the second principal component, which was associated with microclimate refugia availability. During the breeding season, microclimate refugia were relatively limited for three breeding populations (Llanos de Caceres, Campina Sur/La Serena and Castro Verde), compared to the Merida and Vila Fernando populations, which had a broad breeding niche in terms of refugia availability. In the post‐breeding season, however, while both Llanos de Caceres and Campina Sur/La Serena populations seemed to have maintained an almost identical microclimatic niche, the niche of the Castro Verde population expanded in terms of refugia availability. On average, individuals from the Campina Sur/La Serena and Llanos de Caceres populations move farther north during the post‐breeding season than individuals from Castro Verde or any of the other breeding populations studied here. Northern areas are characterized by temperatures up to 10°C lower than the more southern post‐breeding areas (Ramos et al., 2023a). The lower temperatures associated with northern post‐breeding areas are more similar to the temperature range that the individuals are exposed to during breeding, and therefore, the relative thermal strain is lower. Hence, these individuals that travel longer distances to areas with lower temperatures may be less dependent on the availability of microclimate refugia (Ramos et al., 2023a) and are usually niche trackers, a behaviour already documented at the species level for multiple species (Fandos & Tellería, 2020; Somveille et al., 2019). The opposite may be true for birds that do not move as far north, such as the Castro Verde breeding population, as the exposure to relatively higher temperatures within their post‐breeding areas may potentially increase the need for microclimate refugia. Castro Verde is one of the warmest areas within the little bustard's range in the Iberian Peninsula and has had the highest temperature anomaly, reaching 2°C during the breeding season, over the past 30 years (Ramos et al., 2023a). Hence, individuals from this breeding population migrate to areas with more microclimate refugia available (near the coast) and have a more dissimilar post‐breeding niche, as finding shelter from the extreme heat within the area may be necessary to maintain fitness and for their survival. Finally, both the Merida and Vila Fernando populations had broad microclimatic niches with relatively more microclimate refugia available across both the breeding and post‐breeding seasons. The greater refugia availability may allow individuals from these populations to cope with increasing temperatures throughout the summer (the post‐breeding season) and remain in similar areas in both seasons. Thus, these breeding populations have, in general, more similar seasonal niches. These across‐population differences result from diverse individual movement strategies and use of microclimate refugia, which have been associated with increased resilience to environmental change (Gilroy et al., 2016; Ramos et al., 2023b).

While previous studies compared long‐distance migrants to resident birds (Cohen & Jetz, 2023; Gómez et al., 2016; Ponti et al., 2020), this study analysed individual GPS data and the gradient of distances individuals moved, including resident, short‐, medium‐, and long‐distance migrants. At the individual level, the availability of microclimate refugia during the breeding season was negatively associated with the migration distance of male little bustards, with longer distance migrants having less microclimate refugia availability during breeding. Although microclimate refugia availability is dynamic over time, areas with low availability during the breeding season are likely to remain so in post‐breeding (Ramos et al., 2023a). Furthermore, during the breeding season, male little bustards are restricted to lekking areas, which are characterized by open sites that not only allow birds to be seen by conspecifics but also expose them to elevated temperatures due to the lack of features that may shield them from the heat (Ramos et al., 2023a; Silva et al., 2015, 2017). Post‐breeding movements may be the most advantageous strategy for birds experiencing low microclimate refugia availability during their breeding season, allowing them to move to cooler areas or areas with refugia available. Moreover, migration has other benefits such as access to sites with higher food availability (Fandos & Tellería, 2020; Somveille et al., 2019); however, it can also have a potentially detrimental effect on fitness due to its high energetic cost and the increased exposure to potential hazards (Dingle, 2014; Wikelski et al., 2003). On the other hand, individuals that move short distances or are resident are likely exposed to harsh conditions during the warmest period of the year, the post‐breeding season (Fandos & Tellería, 2020). Thus, remaining stationary requires a higher tolerance of sub‐optimal conditions and may involve behavioural and physiological adaptations to cope with unfavourable conditions (Cohen & Jetz, 2023). However, remaining closer to the breeding sites can also be advantageous, as it removes the additional physiological strain of movement, which could otherwise have a negative effect on individual fitness during the subsequent breeding season.

Similarly, migratory distance was also a good predictor of seasonal dissimilarity in the individual's microclimatic niche. Individuals that travel longer distances have low seasonal niche dissimilarity, indicating potential niche tracking. On the other hand, individuals that choose post‐breeding locations closer to the breeding areas may display niche switching strategies (Martínez‐Meyer et al., 2004; Nakazawa et al., 2004). This behaviour has already been documented at the species level for multiple species (Fandos & Tellería, 2020; Somveille et al., 2019). For the studied populations of male little bustards, our results suggest that individuals with less microclimate refugia available during the breeding season move farther away to cooler locations, which can be considered macro‐scale refugia and are characterized by conditions similar to those experienced by the individual during breeding.

While little bustards exhibit high site fidelity during both breeding and post‐breeding seasons, and migratory behaviour is likely a genetic trait (Alonso et al., 2020; Burnside et al., 2017; Villers et al., 2010), migratory behaviour and migratory distance are plastic behaviours that vary between individuals in partially migratory species (Pulido, 2007; Salewski & Bruderer, 2007). As such, a dispersive migration strategy may persist if individuals adopting different movement strategies gain fitness benefits under distinct environmental conditions, and environmental variability and selection do not favour one single strategy (Buchan et al., 2021).

The average NDVI of the last 8 days of each individual's breeding season had no significant effect on the distance travelled by individuals. In the southern Iberian Peninsula, NDVI peaks during April and May and decreases steeply from May to June, as temperature increases (Marcelino et al., 2020). Male little bustards are highly affected by this fluctuation. In areas where NDVI decreases rapidly, they migrate early, whereas areas with higher NDVI allow for a longer breeding period (Ramos et al., 2023b). Towards the end of the breeding season, NDVI is relatively low across all breeding sites, and therefore, all individuals within our study area are likely exposed to similar, low availability of green plants. Furthermore, despite the well‐known importance of green plants in the little bustard diet, during reproduction and periods of food shortage (Bretagnolle et al., 2022; Jiguet, 2002; Silva et al., 2015), recent studies reinforce that arthropods also form an important part of the little bustard diet (Cabodevilla et al., 2021; González del Portillo et al., 2024), indicating that NDVI alone may not capture the full extent of food availability. This could explain why male little bustards, like other bustard species, have a high site fidelity in both the breeding and post‐breeding seasons (Alonso et al., 2020; Burnside et al., 2017), which is also linked with relatively high repeatability of the distance travelled between years (Ramos et al., 2023b).

Nevertheless, caution is needed when extrapolating these findings. During the non‐breeding period, little bustards gather in mixed flocks, including males, females and juvenile individuals (Morales et al., 2022), and thus, all individuals may have similar microclimatic requirements, as well as limitations. However, since only females participate in chick‐rearing, they tend to stay longer in the breeding areas (Schulz, 1986). As the summer progresses and temperatures increase, females and juveniles will potentially experience detrimental environmental conditions, which may be different from those experienced by males. Additionally, the knowledge of female little bustards' movement strategies, such as timings, distance travelled and stopovers, is still largely limited and may differ from the migratory strategies of males, which are better understood (Morales et al., 2022). Thus, we can expect that the post‐breeding microclimatic niche will be similar across males and females; however, during the breeding season, their niches may be significantly different, and therefore, the relationship between the seasons and movement strategies of females may be different from that reported in this study.

This study focuses on birds captured in the Southwest Iberian Peninsula, which exhibit two main migration patterns described for the Iberian populations: resident/sedentary and summer migrants (García de La Morena et al., 2015). However, other dispersive migratory strategies are known within the populations of the Iberian Peninsula (García de La Morena et al., 2015). In particular, populations in the north of Iberia have higher proportions of summer‐winter and winter migrants (Morales et al., 2022), which may display different microclimatic niches and niche similarities between seasons, since they are exposed to lower temperatures across the year (Ramos et al., 2023a). Hence, our findings may be extrapolated to other populations with the same migratory strategies but not to all populations within the Iberian Peninsula.

Finally, the metric used in this study to quantify seasonal differences in microclimatic niches captures a limited representation of a potentially more complex ecological relationship. Seasonal niche dissimilarity, measured as the Euclidean distance between the centroids of the seasonal microclimatic niches, represents the difference between the average position of each niche within the environmental space; however, it does not capture other potentially important niche characteristics, such as niche size or shape. For example, the range of post‐breeding conditions may be completely within the range of breeding conditions, and the two niches may fully overlap, but the centroid distance may be relatively large. Furthermore, niche shifts may be determined by the availability within the environmental space, and this availability is likely to differ seasonally. An individual may select different conditions due to differences in requirements at different stages of the annual cycle or experience seasonal changes in conditions. Thus, accounting for niche position within the total available environmental space would provide a more complete overview of the seasonal niche dynamics. Our approach only partially included this, as the range of microclimatic conditions observed across all individuals and seasons is used to define the range of the two principal component axes. Although quantifying niche overlap while explicitly accounting for the range of conditions that are utilized out of those available would provide a more complete and nuanced overview of seasonal niche dynamics, it is not possible given how computationally intensive microclimate modelling is. For our study, it would require generating the hourly microclimate temperatures at a 30‐m scale across the whole study extent—in this case, the Iberian Peninsula. Our centroid distance approach, while limited, still allows us to reliably quantify dissimilarity between seasonal niches and draw inference on similarity between conditions experienced by individuals in the two seasons, despite not accounting for the available environmental conditions. Our results suggest that migratory distance can determine individual niche similarity between seasons, with short‐distance migrants usually having more dissimilar niches. However, other factors such as niche breadth and breeding latitude are known to influence seasonal niche strategy at the species level (Gómez et al., 2016) and should be considered in future studies. We demonstrated that microclimate refugia availability, particularly during the breeding season, plays a key role in defining microclimatic niche dissimilarity between seasons and can help predict individual movement strategies, which, to our knowledge, has not been demonstrated before.

## AUTHOR CONTRIBUTIONS

Rita F. Ramos, Aldina M. A. Franco and João P. Silva conceived the overall study. João P. Silva was responsible for capturing and tagging the little bustards. Rita F. Ramos prepared the dataset, coded the models and analysed the data, assisted by Karolina Zalewska, James J. Gilroy and Aldina M. A. Franco. Rita F. Ramos and Karolina Zalewska wrote the manuscript assisted by Aldina M. A. Franco with revisions from João P. Silva and James J. Gilroy. All authors contributed critically to the drafts and gave final approval for publication. The study includes authors from different countries, including researchers based in the country where the study was carried out (Portugal). The authors cited all relevant literature published by scientists from the studied region.

## CONFLICT OF INTEREST STATEMENT

The authors declare that there are no conflicts of interest.

## Supporting information


**Supporting Information S1.** Little bustard realised microclimatic niche characterization.
**Figure S1.1.** Correlation circle showing variable contribution to the first (horizontal) and second (vertical) principal components, describing the little bustard's realised microclimatic niche.
**Figure S1.2.** Variable percentage of contribution to the first (a) and second (b) principal components.
**Supporting Information S2.** Realised microclimatic niche for the five breeding areas.
**Figure S2.** Realised microclimatic niche of each breeding population during breeding (a) and post‐breeding (b) seasons. Each population is represented by a different colour. Black outline shows the realised microclimatic niche across all populations and seasons. Dashed grey outline shows realised microclimatic niche of all populations for breeding (a) and post‐breeding (b).
**Supporting Information S3.** Test of breeding population niche size based on number of individuals.
**Figure S3.** Variation in the percentage of overlap of the seasonal niche produced using randomly selected five individuals per breeding area and the corresponding total seasonal niche. Orange and light‐blue boxplots represent the breeding and post‐breeding seasons, respectively. Red rhombuses represent the percentage of overlap obtained when using all the individuals of each population (percentages given in the manuscript).
**Supporting Information S4.** Information of distance travelled and realised microclimatic niche.
**Table S4.** The distance travelled, seasonal niche dissimilarity, number of GPS points collected, used area (calculated as the 90% kernel of all relocations), and the percentage of overlap of the realised microclimatic niche with the population seasonal niche (% ind – pop.) and total niche (% ind. – total), for each individual in each year, separately for the breeding and post‐breeding seasons, as well as the population‐level overlap of the seasonal niche with the total niche of the corresponding season (% pop. – total).
**Supporting Information S5.** Distance travelled and niche dissimilarity models.
**Figure S5.1.** Parameter estimates from the LMM explaining distance travelled using the scaled *x* (Temperature) and *y* (Microclimate refugia availability) centroid coordinate of the individual breeding niche, and the average NDVI of the last eight days of the individual's breeding season. The breeding population (breeding site) is included as a random effect.
**Figure S5.2.** Parameter estimates from the LMM explaining niche dissimilarity between seasons using the percentage of overlap of individual breeding niche with the population breeding niche (% breeding season occupied niche), the scaled *x* (Temperature) and *y* (Microclimate refugia availability) centroid coordinate of the individual breeding niche, and the distance travelled. The breeding population (breeding site) is included as a random effect.

## Data Availability

Data available from the Dryad Digital Repository: https://doi.org/10.5061/dryad.280gb5n38 (Ramos et al., 2025).
