# Supplementary material for: Microclimate refugia shape microclimatic niches and predict individual variability in post‐breeding migration in a partially migratory species
Source: J Anim Ecol. 2025 Sep 28;94(12):2527–41. doi: 10.1111/1365-2656.70147 (PMC12673241; doi:10.1111/1365-2656.70147)
Supplement: Supplementary file 1 — Supporting Information S1. Little bustard realised microclimatic niche characterization. Figure S1.1. Correlation circle showing variable contribution to the first (horizontal) and second (vertical) principal components, describing the little bustard's realised microclimatic niche. Figure S1.2. Variable percentage of contribution to the first (a) and second (b) principal components. Supporting Information S2. Realised microclimatic niche for the five breeding areas. Figure S2. Realised microclimatic niche of each breeding population during breeding (a) and post‐breeding (b) seasons. Each population is represented by a different colour. Black outline shows the realised microclimatic niche across all populations and seasons. Dashed grey outline shows realised microclimatic niche of all populations for breeding (a) and post‐breeding (b). Supporting Information S3. Test of breeding population niche size based on number of individuals. Figure S3. Variation in the percentage of overlap of the seasonal niche produced using randomly selected five individuals per breeding area and the corresponding total seasonal niche. Orange and light‐blue boxplots represent the breeding and post‐breeding seasons, respectively. Red rhombuses represent the percentage of overlap obtained when using all the individuals of each population (percentages given in the manuscript). Supporting Information S4. Information of distance travelled and realised microclimatic niche. Table S4. The distance travelled, seasonal niche dissimilarity, number of GPS points collected, used area (calculated as the 90% kernel of all relocations), and the percentage of overlap of the realised microclimatic niche with the population seasonal niche (% ind – pop.) and total niche (% ind. – total), for each individual in each year, separately for the breeding and post‐breeding seasons, as well as the population‐level overlap of the seasonal niche with the total niche of the corresponding season (% pop. – total). Supporti [file JANE-94-2527-s001.docx]

**Microclimate refugia availability shapes microclimatic niches and predicts the dispersive migration strategies of individuals**

**S1 – Little bustard realised microclimatic niche characterization**


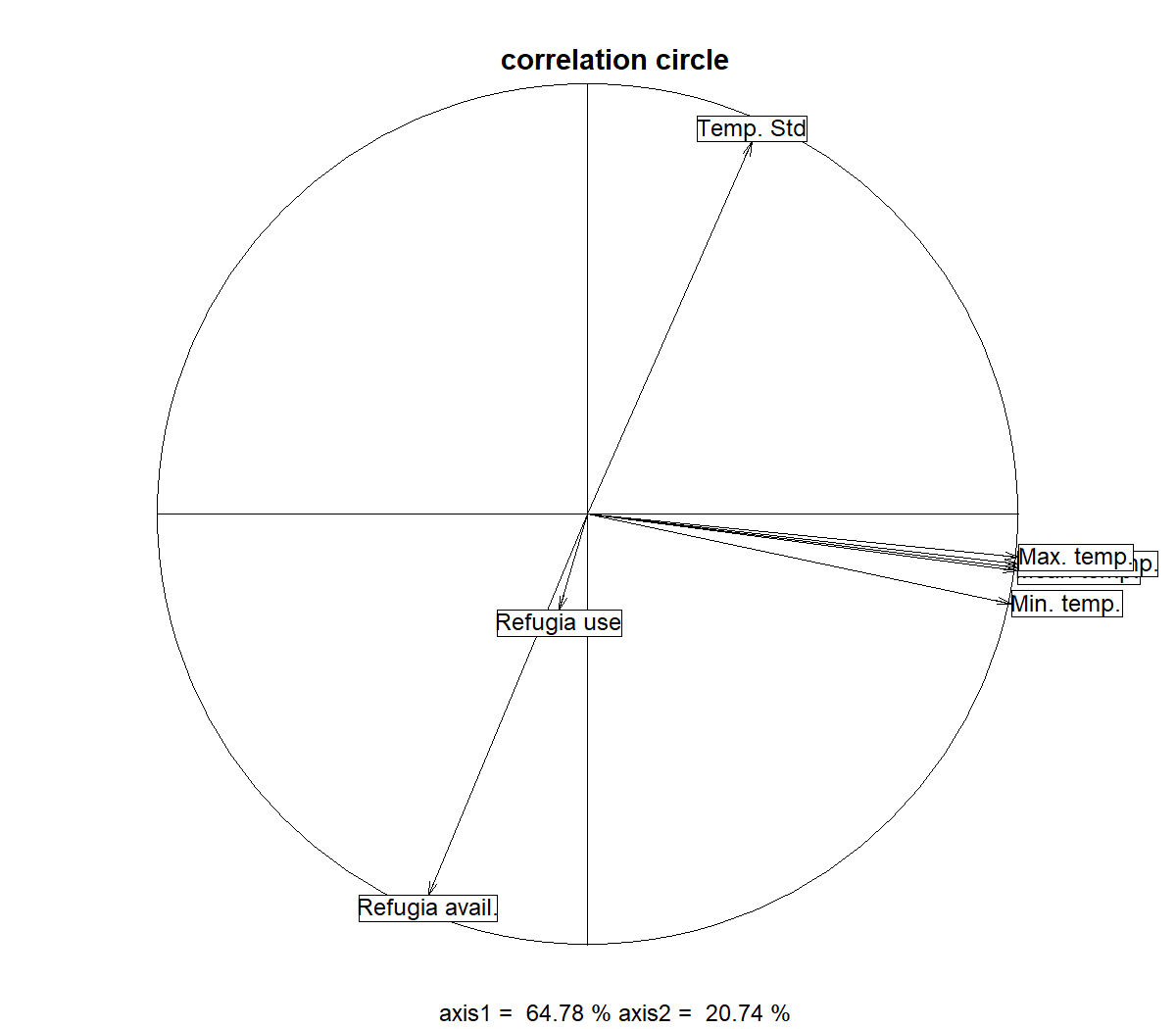


**Figure S1.1 –** Correlation circle showing variable contribution to the first (horizontal) and second (vertical) principal components, describing the little bustard’s realised microclimatic niche

Negative values of ‘Refugia Use’ and ‘Refugia Availability’ variables indicate microclimate refugia use and availability, as it indicates point temperatures cooler that the median of the buffer (Ramos et al., 2023a). Hence, the positive values of Axis 2 represent greater refugia use and refugia availability, and higher temperature standard deviation within the buffer.

| **a)** | 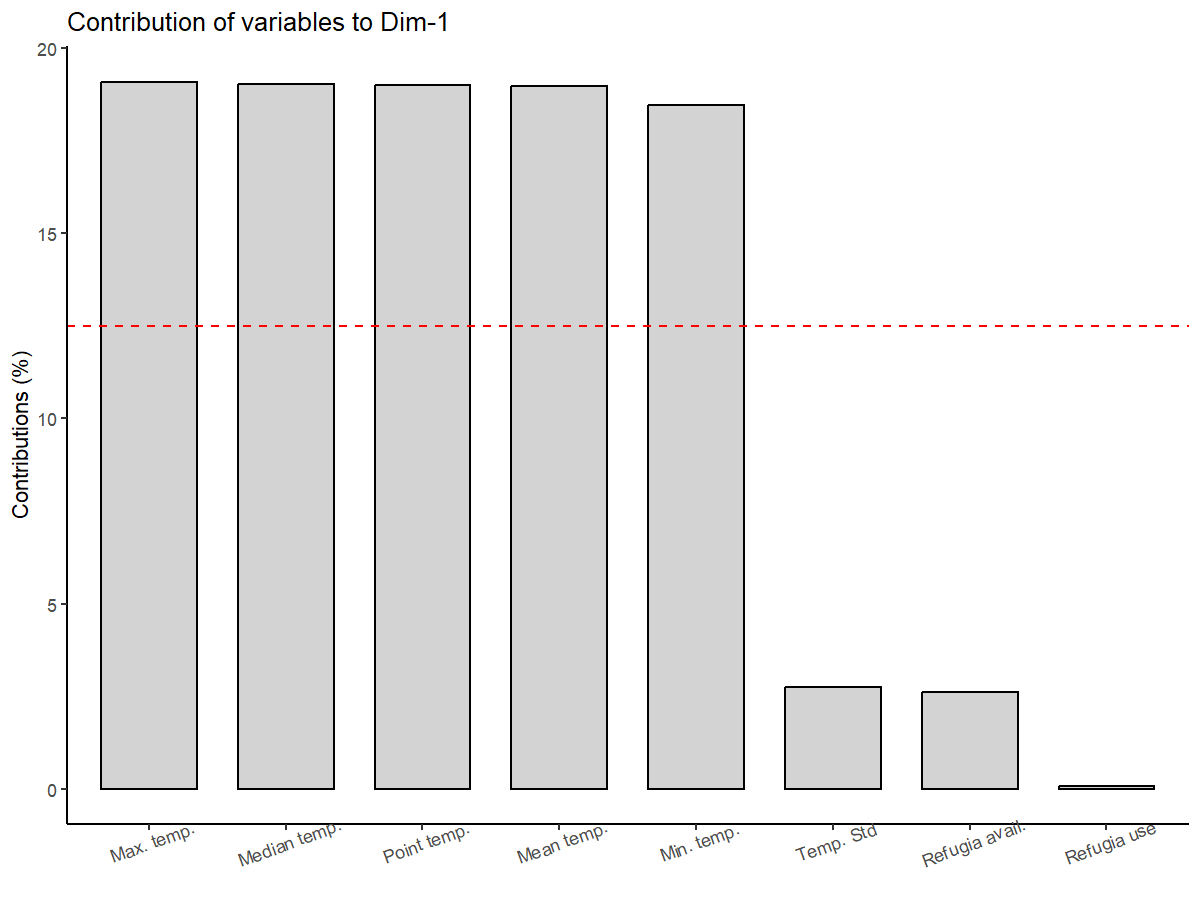 |
| --- | --- |
| **b)** | 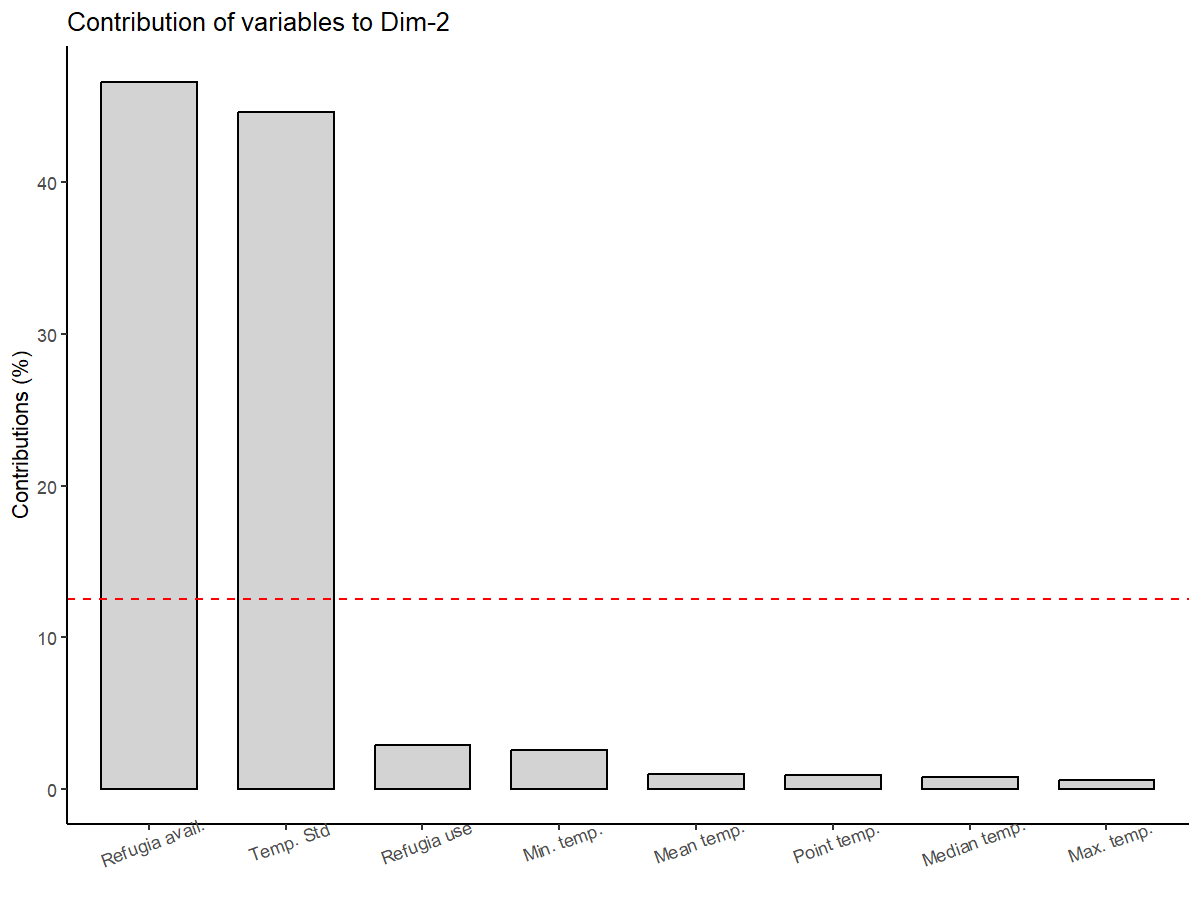 |

**Figure S1.2** **–** Variable percentage of contribution to the first (a) and second (b) principal components.

**S2 – Realised microclimatic niche for the five breeding areas**


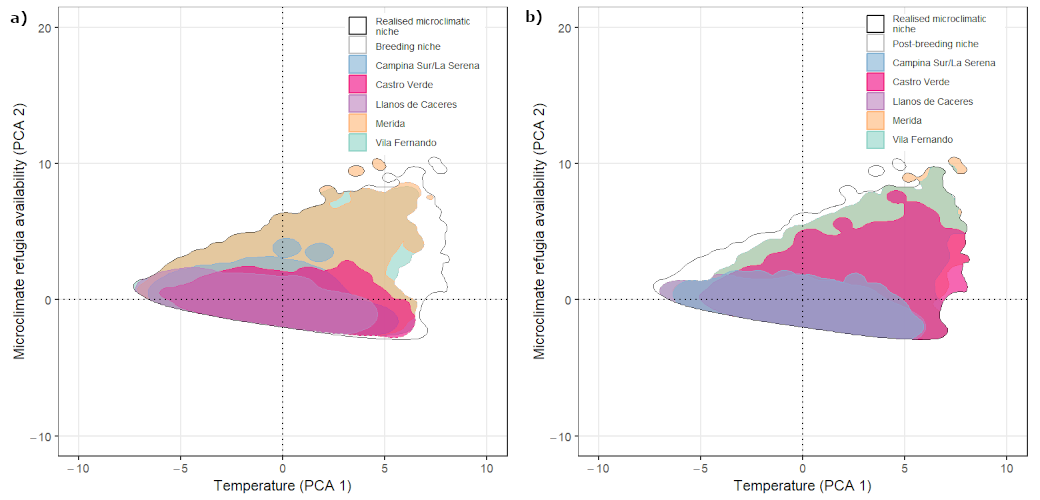
**Figure S2 –** Realised microclimatic niche of each breeding population during breeding (a) and post-breeding (b) seasons. Each population is represented by a different colour. Black outline shows the realised microclimatic niche across all populations and seasons. Dashed grey outline shows realised microclimatic niche of all populations for breeding (a) and post-breeding (b).

**S3 – Test of breeding population niche size based on number of individuals**

Two of the five breeding populations have few individuals tagged. While the breeding used areas of the two populations were the second and third largest across all populations in the study (Table 2), their breeding niches were smaller than 50% of the total niche. This raised the question if such a small sample size could be representative of the population.

To address this question, for all breeding populations with larger sample sizes (Castro Verde: 26 individuals, Vila Fernando: 23 individuals and Merida: 9 individuals), we randomly selected five individuals, produced the seasonal microclimatic niche for the population based only on those individuals, then calculated the percentage of overlap with the total niche of the corresponding season. We repeated this process for all possible combinations of 5 individuals within each breeding populations for each season, up to a maximum of 10,000 combinations, due to memory and processing limitations.

The results obtained are presented in Figure S3, where the red rhombuses represent the percentage of overlap obtained running the analysis for all the individuals in each population. Since the rhombuses are within the boxplot ranges in the upper quadrant, we do not expect that the two populations with the relatively lower sample sizes (Llanos de Caceres and Campina Sur/La Serena) are underrepresented.


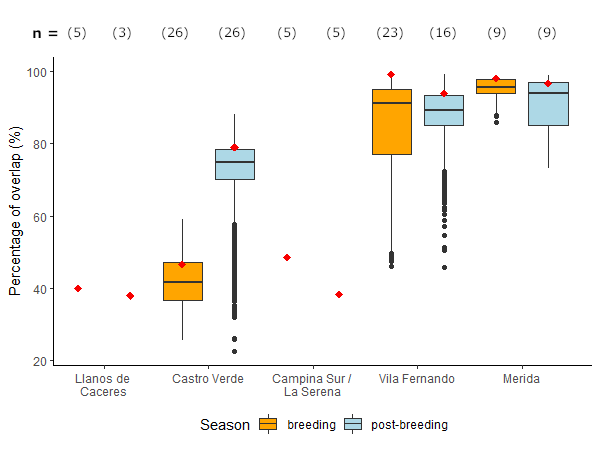


**Figure S3** **–** Variation in the percentage of overlap of the seasonal niche produced using randomly selected five individuals per breeding area and the corresponding total seasonal niche. Orange and light-blue boxplots represent the breeding and post-breeding seasons, respectively. Red rhombuses represent the percentage of overlap obtained when using all the individuals of each population (percentages given in the manuscript).

**S4 – Information of distance travelled and realised microclimatic niche**

**Table S4 -** The distance travelled, seasonal niche dissimilarity, number of GPS points collected, used area (calculated as the 90% kernel of all relocations), and the percentage of overlap of the realised microclimatic niche with the population seasonal niche (% ind – pop.) and total niche (% ind. - total), for each individual in each year, separately for the breeding and post-breeding seasons, as well as the population-level overlap of the seasonal niche with the total niche of the corresponding season (% pop. – total).

| Breeding population | Year | Individual ID | Post-breeding distances travelled (km) | Niche dissimilarity | Breeding season | | | | |
| --- | --- | --- | --- | --- | --- | --- | --- | --- | --- |
|  |  |  |  |  | **% pop. - total** | **% ind. – pop.** | **% ind. - total** | **Num. GPS points** | **Used areas (ha)** |
| Campina Sur / La Serena | 2015 | Boot7 | 27.04 | 0.35 | 33.77 | 100.00 | 33.77 | 904 | 46.75 |
|  |  | Boot8 | 347.08 | 1.33 |  | 97.49 | 32.92 | 1027 | 35.39 |
|  | 2019 | Cabeza_1 | 161.64 | 1.24 | 52.32 | 60.57 | 31.69 | 195 | 207.87 |
|  |  | Cabeza_2 | 71.18 | 1.78 |  | 55.55 | 29.06 | 337 | 610.17 |
|  |  | Cabeza_3 | 52.00 | 1.16 |  | 100.00 | 52.32 | 707 | 275.00 |
| Castro Verde | 2009 | PTT_91469_1 | 165.77 | 0.83 | 33.27 | 98.03 | 32.62 | 108 | 237.51 |
|  |  | PTT_91470_1 | 77.17 | 0.81 |  | 100.00 | 33.27 | 84 | 298.95 |
|  |  | PTT_91473_1 | 111.12 | 0.95 |  | 100.00 | 33.27 | 114 | 73.24 |
|  |  | PTT_91481_1 | 56.85 | 2.77 |  | 99.03 | 32.95 | 199 | 31.83 |
|  | 2010 | PTT_91469_2 | 28.75 | 2.36 | 36.30 | 100.00 | 36.30 | 360 | 62.49 |
|  |  | PTT_91470_1 | 77.74 | 1.40 |  | 100.00 | 36.30 | 355 | 142.15 |
|  |  | PTT_91471_2 | 39.39 | 3.98 |  | 83.60 | 30.35 | 133 | 68.51 |
|  |  | PTT_91475_2 | 33.56 | 3.29 |  | 99.74 | 36.21 | 312 | 141.60 |
|  |  | PTT_91481_1 | 37.54 | 3.33 |  | 100.00 | 36.30 | 354 | 476.09 |
|  | 2011 | PTT_91469_2 | 30.52 | 2.09 | 34.11 | 100.00 | 34.11 | 436 | 64.86 |
|  |  | PTT_91470_2 | 69.30 | 1.88 |  | 91.17 | 31.10 | 95 | 4.55 |
|  |  | PTT_91471_2 | 32.46 | 1.89 |  | 99.96 | 34.09 | 312 | 47.83 |
|  |  | PTT_91473_3 | 30.12 | 0.73 |  | 100.00 | 34.11 | 418 | 111.15 |
|  |  | PTT_91475_3 | 35.38 | 2.94 |  | 80.33 | 27.40 | 238 | 90.90 |
|  |  | PTT_91476_4 | 77.68 | 2.00 |  | 90.92 | 31.01 | 96 | 642.48 |
|  |  | PTT_91478_5 | 31.40 | 1.98 |  | 100.00 | 34.11 | 355 | 140.83 |
|  |  | PTT_91482_2 | 148.75 | 0.90 |  | 99.66 | 33.99 | 465 | 278.08 |
|  | 2012 | PTT_91470_2 | 1.66 | 1.36 | 38.57 | 42.84 | 16.52 | 444 | 257.08 |
|  |  | PTT_91476_4 | 78.34 | 2.50 |  | 100.00 | 38.57 | 126 | 82.72 |
|  | 2015 | Acc7 | 155.86 | 1.99 | 58.64 | 100.00 | 58.64 | 155 | 270.08 |
|  | 2017 | LB_01_pt | 48.02 | 0.76 | 51.01 | 100.00 | 51.01 | 222 | 4.86 |
|  | 2019 | lb_pt_2 | 185.56 | 1.02 | 32.03 | 95.53 | 30.60 | 199 | 1069.08 |
|  |  | lb_pt_3 | 45.57 | 1.75 |  | 86.81 | 27.80 | 708 | 41.37 |
|  |  | lb_pt_4 | 42.19 | 2.41 |  | 79.50 | 25.46 | 652 | 180.64 |
|  |  | lb_pt_5 | 34.64 | 2.51 |  | 81.42 | 26.08 | 677 | 41.65 |
|  |  | lb_pt_6 | 35.46 | 2.50 |  | 89.47 | 28.65 | 393 | 96.07 |
| Llanos Caceres | 2014 | LB-4 | 70.27 | 0.83 | 34.42 | 100.00 | 34.42 | 936 | 117.01 |
|  | 2015 | Acc4 | 201.27 | 0.38 | 34.34 | 100.00 | 34.34 | 253 | 135.98 |
|  |  | Boot4 | 55.40 | 0.68 |  | 99.91 | 34.31 | 220 | 213.88 |
|  |  | Boot5 | 112.61 | 0.99 |  | 99.96 | 34.32 | 193 | 3.00 |
|  | 2016 | Boot4 | 6.43 | NA | 39.63 | 98.06 | 38.86 | 767 | 23.49 |
|  |  | Boot5 | 87.37 | NA |  | 79.96 | 31.69 | 295 | 18.20 |
| Merida | 2015 | Boot3 | 18.00 | 0.96 | 95.20 | 100.00 | 95.20 | 544 | 724.79 |
|  | 2016 | Boot3 | 23.90 | 1.02 | 95.31 | 100.00 | 95.31 | 1108 | 6607.78 |
|  | 2017 | Boot3 | 15.01 | 1.42 | 94.05 | 94.73 | 89.09 | 404 | 632.81 |
|  |  | LB_09_sp | NA | 1.57 |  | 71.56 | 67.30 | 789 | 38.86 |
|  |  | LB_11_sp | 6.12 | 2.34 |  | 75.28 | 70.80 | 620 | 4.97 |
|  |  | LB_12_sp | 7.61 | 1.39 |  | 86.40 | 81.26 | 1397 | 95.03 |
|  | 2018 | LB_09_sp | 18.10 | 2.53 | 77.10 | 64.38 | 49.64 | 914 | 29.00 |
|  |  | LB_Mirandilla | 15.58 | 1.63 |  | 99.23 | 76.51 | 628 | 913.74 |
|  | 2019 | LB_09_sp | 18.32 | 1.85 | 70.33 | 100.00 | 70.33 | 793 | 937.92 |
| Vila Fernando | 2009 | PTT_91471_1 | 232.79 | 0.21 | 76.98 | 72.43 | 55.75 | 371 | 52.35 |
|  |  | PTT_91472 | 9.16 | NA |  | 97.65 | 75.17 | 288 | 955.85 |
|  |  | PTT_91475_1 | 14.78 | 1.76 |  | 73.37 | 56.48 | 270 | 80.89 |
|  |  | PTT_91479 | 55.76 | 2.96 |  | 63.55 | 48.92 | 265 | 23.16 |
|  |  | PTT_91480 | 15.40 | 2.82 |  | 65.63 | 50.52 | 300 | 1152.61 |
|  |  | PTT_91482_1 | 1.36 | 1.85 |  | 92.76 | 71.40 | 439 | 751.23 |
|  | 2010 | PTT_91479 | NA | 2.31 | 74.60 | 98.24 | 73.29 | 458 | 13429.45 |
|  |  | PTT_91480 | NA | 2.97 |  | 76.67 | 57.20 | 373 | 240.07 |
|  |  | PTT_91482_1 | 1.59 | 2.73 |  | 88.23 | 65.82 | 437 | 53.21 |
|  | 2011 | PTT_91480 | 14.99 | 1.57 | 53.76 | 100.00 | 53.76 | 351 | 202.07 |
|  | 2012 | PTT_91480 | NA | NA | 52.09 | 100.00 | 52.09 | 360 | 25.80 |
|  | 2014 | LB-1 | 10.71 | 1.78 | 35.33 | 93.58 | 33.06 | 157 | 98.70 |
|  |  | LB-3 | 18.06 | 1.24 |  | 99.99 | 35.33 | 209 | 94.26 |
|  | 2015 | LB-1 | 3.86 | 2.41 | 91.58 | 55.88 | 51.17 | 71 | 211.68 |
|  |  | Acc10 | NA | NA |  | 60.02 | 54.97 | 183 | 136.86 |
|  |  | Acc11 | 20.80 | 1.52 |  | 81.08 | 74.25 | 330 | 93.52 |
|  |  | Acc13 | 143.83 | 1.25 |  | 54.72 | 50.11 | 130 | 147.52 |
|  |  | Acc14 | NA | NA |  | 56.64 | 51.87 | 164 | 31.61 |
|  |  | Acc8 | 0.30 | 2.66 |  | 98.94 | 90.61 | 1571 | 82.17 |
|  |  | Boot6 | 9.66 | 1.11 |  | 70.06 | 64.16 | 1243 | 308.15 |
|  | 2016 | LB-1 | 9.25 | 2.16 | 96.17 | 49.95 | 48.03 | 325 | 71.63 |
|  |  | Acc11 | 41.29 | 1.02 |  | 100.00 | 96.17 | 709 | 151.48 |
|  |  | Acc13 | 117.58 | 1.30 |  | 45.10 | 43.38 | 207 | 547.26 |
|  | 2017 | LB-1 | 4.41 | 1.50 | 69.14 | 95.90 | 66.30 | 265 | 371.64 |
|  |  | LB_16_pt | 420.63 | 2.65 |  | 60.90 | 42.10 | 248 | 157.98 |

| Breeding population | Year | Individual ID | Post-breeding season | | | | |
| --- | --- | --- | --- | --- | --- | --- | --- |
|  |  |  | **% pop. - total** | **% ind. – pop.** | **% ind. - total** | **Num. GPS points** | **Used areas (ha)** |
| Campina Sur / La Serena | 2015 | Boot7 | 39.15 | 100.00 | 39.14 | 966 | 752.85 |
|  |  | Boot8 |  | 46.10 | 18.04 | 792 | 75.62 |
|  | 2019 | Cabeza_1 | 34.44 | 87.51 | 30.14 | 2466 | 1311.14 |
|  |  | Cabeza_2 |  | 92.33 | 31.80 | 735 | 2051.72 |
|  |  | Cabeza_3 |  | 99.62 | 34.31 | 777 | 10378.00 |
| Castro Verde | 2009 | PTT_91469_1 | 63.33 | 18.61 | 11.79 | 410 | 8939.74 |
|  |  | PTT_91470_1 |  | 24.45 | 15.48 | 285 | 557.04 |
|  |  | PTT_91473_1 |  | 59.39 | 37.61 | 300 | NA |
|  |  | PTT_91481_1 |  | 100.00 | 63.33 | 427 | 4196.58 |
|  | 2010 | PTT_91469_2 | 75.37 | 75.73 | 57.08 | 419 | 589.29 |
|  |  | PTT_91470_1 |  | 33.49 | 25.24 | 432 | 418.87 |
|  |  | PTT_91471_2 |  | 99.64 | 75.10 | 395 | 3247.18 |
|  |  | PTT_91475_2 |  | 97.68 | 73.62 | 396 | 671.46 |
|  |  | PTT_91481_1 |  | 99.71 | 75.15 | 156 | 1215.26 |
|  | 2011 | PTT_91469_2 | 77.82 | 92.14 | 71.70 | 425 | 161.52 |
|  |  | PTT_91470_2 |  | 31.71 | 24.68 | 435 | 608.78 |
|  |  | PTT_91471_2 |  | 87.50 | 68.09 | 410 | 19002.70 |
|  |  | PTT_91473_3 |  | 48.44 | 37.69 | 431 | 946.57 |
|  |  | PTT_91475_3 |  | 98.63 | 76.75 | 391 | 2055.12 |
|  |  | PTT_91476_4 |  | 87.31 | 67.94 | 422 | 13222.87 |
|  |  | PTT_91478_5 |  | 91.08 | 70.88 | 299 | 10338.55 |
|  |  | PTT_91482_2 |  | 54.92 | 42.74 | 375 | NA |
|  | 2012 | PTT_91470_2 | 59.61 | 36.75 | 21.91 | 286 | 960.06 |
|  |  | PTT_91476_4 |  | 93.02 | 55.45 | 325 | 9037.51 |
|  | 2015 | Acc7 | 15.85 | 100.00 | 15.85 | 260 | 2595.08 |
|  | 2017 | LB_01_pt | 9.65 | 100.00 | 9.65 | 848 | 148.24 |
|  | 2019 | lb_pt_2 | 67.65 | 13.97 | 9.45 | 651 | 15436.83 |
|  |  | lb_pt_3 |  | 77.94 | 52.73 | 398 | 188.37 |
|  |  | lb_pt_4 |  | 92.59 | 62.64 | 792 | 16709.39 |
|  |  | lb_pt_5 |  | 90.12 | 60.97 | 729 | 2727.75 |
|  |  | lb_pt_6 |  | 97.37 | 65.88 | 507 | 1086.79 |
| Llanos Caceres | 2014 | LB-4 | 35.72 | 100.00 | 35.72 | 884 | 139.85 |
|  | 2015 | Acc4 | 35.66 | 85.67 | 30.55 | 584 | NA |
|  |  | Boot4 |  | 94.02 | 33.53 | 816 | 132.36 |
|  |  | Boot5 |  | 92.69 | 33.05 | 827 | 2506.68 |
|  | 2016 | Boot4 | NA | NA | NA | NA | NA |
|  |  | Boot5 |  | NA | NA | NA | NA |
| Merida | 2015 | Boot3 | 77.60 | 100.00 | 77.60 | 455 | 9200.68 |
|  | 2016 | Boot3 | 77.03 | 100.00 | 77.03 | 443 | 17451.85 |
|  | 2017 | Boot3 | 95.30 | 94.07 | 89.65 | 729 | 3122.45 |
|  |  | LB_09_sp |  | 68.60 | 65.38 | 1195 | 71.87 |
|  |  | LB_11_sp |  | 94.69 | 90.24 | 1498 | 37310.36 |
|  |  | LB_12_sp |  | 72.38 | 68.98 | 1048 | 661.65 |
|  | 2018 | LB_09_sp | 88.55 | 84.35 | 74.69 | 1263 | 468.50 |
|  |  | LB_Mirandilla |  | 99.51 | 88.11 | 416 | 14461.87 |
|  | 2019 | LB_09_sp | 59.48 | 100.00 | 59.48 | 1328 | 137.49 |
| Vila Fernando | 2009 | PTT_91471_1 | 91.21 | 52.45 | 47.85 | 79 | 1032.98 |
|  |  | PTT_91472 |  | NA | NA | NA | NA |
|  |  | PTT_91475_1 |  | 53.29 | 48.61 | 203 | 202.23 |
|  |  | PTT_91479 |  | 98.56 | 89.90 | 349 | 16810.64 |
|  |  | PTT_91480 |  | 96.03 | 87.59 | 378 | 35471.79 |
|  |  | PTT_91482_1 |  | 99.09 | 90.39 | 290 | 319.01 |
|  | 2010 | PTT_91479 | 96.83 | 99.99 | 96.82 | 388 | 17110.51 |
|  |  | PTT_91480 |  | 89.83 | 86.98 | 399 | 1178.77 |
|  |  | PTT_91482_1 |  | 98.85 | 95.72 | 283 | 798.31 |
|  | 2011 | PTT_91480 | 73.53 | 100.00 | 73.53 | 348 | NA |
|  | 2012 | PTT_91480 | NA | NA | NA | NA | NA |
|  | 2014 | LB-1 | 46.83 | 99.93 | 46.79 | 205 | 1876.78 |
|  |  | LB-3 |  | 78.54 | 36.77 | 250 | 18352.56 |
|  | 2015 | LB-1 | 78.92 | 92.96 | 73.37 | 146 | 340.83 |
|  |  | Acc10 |  | NA | NA | NA | NA |
|  |  | Acc11 |  | 98.31 | 77.59 | 313 | 6411.36 |
|  |  | Acc13 |  | 11.51 | 9.08 | 164 | 1141.91 |
|  |  | Acc14 |  | NA | NA | NA | NA |
|  |  | Acc8 |  | 36.65 | 28.93 | 304 | 56.97 |
|  |  | Boot6 |  | 94.40 | 74.51 | 1385 | 3153.97 |
|  | 2016 | LB-1 | 96.49 | 73.46 | 70.88 | 66 | 3622.11 |
|  |  | Acc11 |  | 100.00 | 96.49 | 335 | NA |
|  |  | Acc13 |  | 41.34 | 39.89 | 18 | NA |
|  | 2017 | LB-1 | 80.07 | 92.98 | 74.44 | 242 | 12531.98 |
|  |  | LB_16_pt |  | 92.50 | 74.06 | 442 | NA |

**S5 – Distance travelled and niche dissimilarity models**

The parameter estimates for the distance travelled and niche dissimilarity models are presented below. There seems to be no variability in distance travelled (Figure S5.1) and niche dissimilarity (Figure S5.2) across populations. However, the breeding site (population) random effect was retained was included in the models to account for other factors differentiating populations that we could not measure with the fix effects of the model.


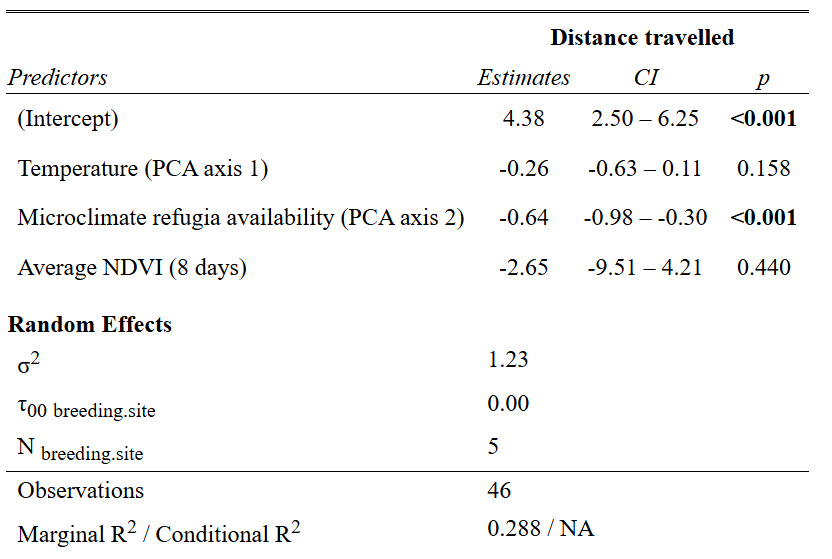


**Figure S5.1 –** Parameter estimates from the LMM explaining distance travelled using the scaled x (Temperature) and y (Microclimate refugia availability) centroid coordinate of the individual breeding niche, and the average NDVI of the last eight days of the individual’s breeding season. The breeding population (breeding site) is included as a random effect.


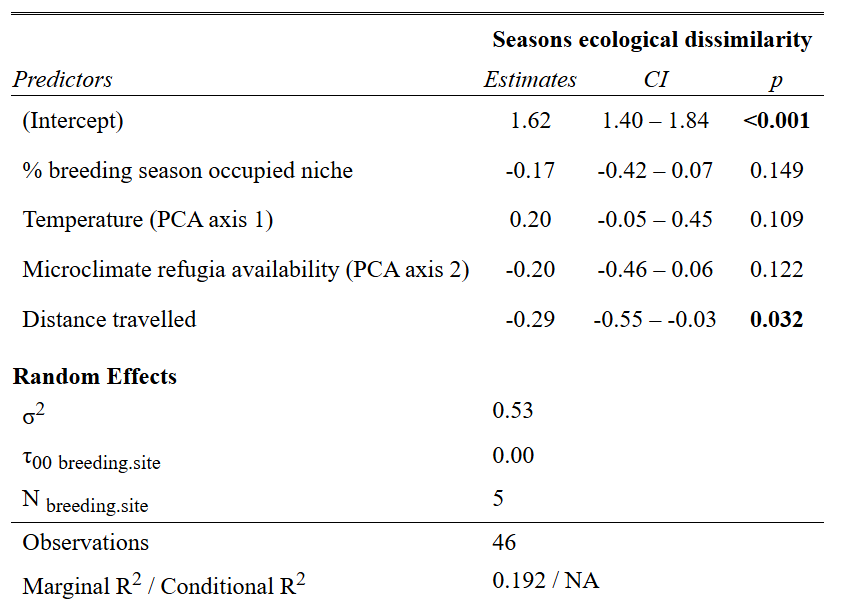


**Figure S5.2 –** Parameter estimates from the LMM explaining niche dissimilarity between seasons using the percentage of overlap of individual breeding niche with the population breeding niche (% breeding season occupied niche), the scaled x (Temperature) and y (Microclimate refugia availability) centroid coordinate of the individual breeding niche, and the distance travelled. The breeding population (breeding site) is included as a random effect.
